# High prevalence of human coronaviruses in rural and urban Ghana in the post-COVID-19 era

**DOI:** 10.3389/fepid.2026.1874054

**Published:** 2026-08-10

**Authors:** Elvis Suatey Lomotey, Martin Montiel-Ruiz, Daniel Adjei Odumang, Elizabeth Obeng-Aboagye, Florence Biamah Amakye, Joseph Annan Hayford, Bernard Aniakwo Logonia, Katharina Röltgen, Irene Owusu Donkor

**Affiliations:** 1 Department of Epidemiology, Noguchi Memorial Institute for Medical Research, University of Ghana, Legon, Accra, Ghana; 2 West African Centre for Cell Biology of Infectious Pathogens, College of Basic and Applied Sciences, University of Ghana, Legon, Accra, Ghana; 3 Department of Parasitology, Noguchi Memorial Institute for Medical Research, University of Ghana, Legon, Accra, Ghana; 4 Department of Medical Parasitology and Infection Biology, Swiss Tropical and Public Health Institute, Allschwil, Switzerland; 5 University of Basel, Basel, Switzerland

**Keywords:** endemic human coronaviruses, epidemiology, Ghana, meso scale discovery, RT-qPCR, SARS-CoV-2, serology, West Africa

## Abstract

The prevalence and distribution of endemic human coronaviruses (HCoVs) in Ghana remain poorly characterized, particularly in the post-COVID-19 pandemic era. We conducted a cross-sectional molecular and serological survey of 1,000 individuals from five rural and five urban communities in Ghana to assess the prevalence of HCoVs. Reverse transcriptase real-time PCR (RT-qPCR) assays targeted SARS-CoV-2, HCoV-OC43, HCoV-HKU1, HCoV-NL63, and HCoV-229E, while serological assays evaluated IgG antibody responses against SARS-CoV-2, SARS-CoV-1, MERS-CoV, HCoV-OC43, HCoV-HKU1, HCoV-NL63, and HCoV-229E Spike proteins. A broad pan-coronavirus RT-qPCR assay detected HCoV RNA in 6.7% of participants, with SARS-CoV-2 as the most frequent, followed by HCoV-229E, HCoV-HKU1, and HCoV-OC43; HCoV-NL63 was not detected. Serological analyses revealed widespread exposure to SARS-CoV-2, HCoV-OC43, HCoV-229E, and HCoV-HKU1, with a low seroprevalence for HCoV-NL63. Our findings demonstrate ongoing co-circulation of multiple HCoVs in both rural and urban settings in Ghana post Covid-19, suggesting sustained community transmission, increased potential for co-infection, and the need for continued surveillance of endemic and emerging respiratory viruses. The data provides rare insights into the epidemiology of HCoVs in West Africa.

## Introduction

Seven human coronaviruses have been identified to date, including four endemic species (HCoV-OC43, HCoV-HKU1, HCoV-NL63, and HCoV-229E) that circulate globally, and three highly pathogenic viruses (SARS-CoV-1, MERS-CoV, and SARS-CoV-2) responsible for severe disease outbreaks. The epidemiology of the endemic species has been well characterized in the Northern hemisphere (1, 2), but data from West Africa remain limited, leaving important gaps in our understanding of their prevalence, circulation patterns, and seasonality.

Public health interventions and behavioral changes implemented during the COVID-19 pandemic have reportedly altered the global transmission dynamics of respiratory viruses, such as influenza viruses (3), and may similarly have influenced the epidemiology of HCoVs. SARS-CoV-2 itself has now also largely transitioned to an endemic pathogen, being both widespread and persistent, adding to the spectrum of respiratory infections that are endemic worldwide (4).

In Ghana, circulation of the four endemic HCoVs was documented prior to the pandemic, with marked seasonal variation in prevalence between the harmattan and wet seasons (5). Updated post-COVID-19 pandemic prevalence data from Ghana provide a critical opportunity to assess the current circulation of HCoVs and to establish a region-specific baseline to guide future surveillance efforts. To address this, we conducted a cross-sectional survey of 1,000 individuals from ten communities—five rural and five urban—across the Greater Accra and Eastern Regions of Ghana. We used pan-coronavirus and HCoV-specific RT-qPCR assays to detect viral RNA as a measure of active infection, and multiplex serological assays to assess prior exposure.

## Methods

### Study design

**Figure 1:**
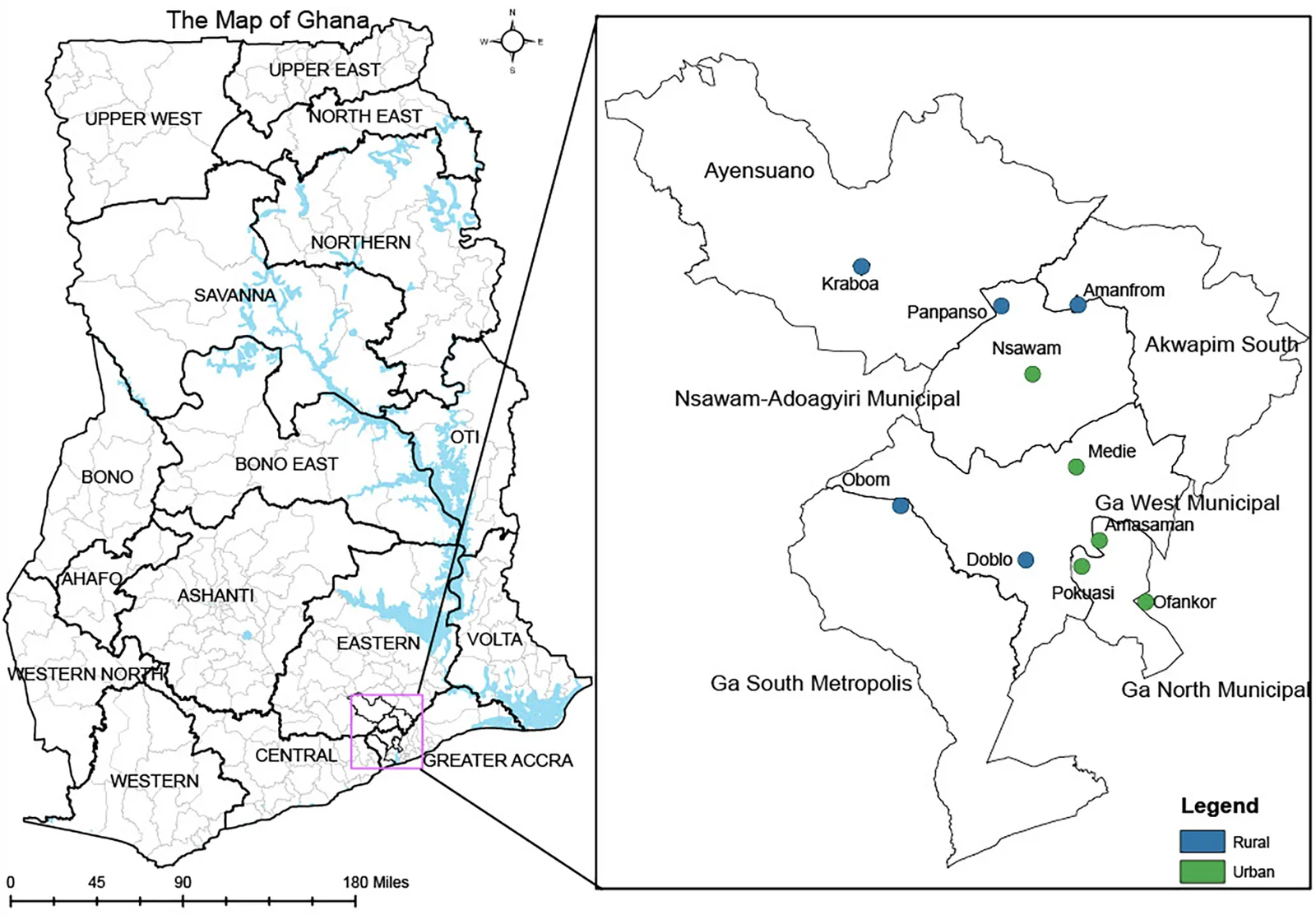
Map of Ghana showing the distribution of the rural and urban study communities. Blue labels denote rural communities (Kraboa, Panpanso, Amanfrom, Doblo, and Obom) and green labels denote urban communities (Nsawam, Medie, Amasaman, Pokuase, and Ofankor) in the Greater Accra and Eastern Regions.

This was a cross-sectional molecular and serological survey conducted between November and December 2022 in the Greater Accra and Eastern Regions of Ghana. The data presented here forms part of a larger coronavirus study conducted in rural and urban communities, with further methodological details described in (6). Briefly, urban settlements (Nsawam, Medie, Amasaman, Pokuase, and Ofankor) situated along the main highway between Accra-Kumasi, characterized by higher population density and greater infrastructure were selected for inclusion in the study. Selected rural communities were Kraboa, Panpanso, Amanfrom, Doblo, and Obom which consist of remote settlements with scattered populations and limited access to basic infrastructure (Figure 1). A total of 1,000 participants were randomly enrolled, with all individuals above two years of age eligible for inclusion irrespective of symptom status. Participant recruitment and sample collection were community-based and conducted through household visits. The cohort included 654 women and 346 men, ranging in age from three to 90 years. Written informed consent was obtained from all participants prior to enrolment, with legally authorized representatives providing consent on behalf of minors.

### Laboratory analysis

Naso-/oropharyngeal swabs were collected from each participant and placed into a single tube containing viral transport medium, in accordance with standard operating procedures (7). Blood was collected in cell preparation tubes (CPT, Becton Dickinson) with sodium citrate and centrifuged at 1,800×*g* for 30 min at room temperature. Swab and plasma samples were aliquoted and stored at −80 °C on the same day. Viral RNA was extracted from naso-/oropharyngeal swabs using the QIAamp Viral RNA Mini Kit (Qiagen, Hilden, Germany), according to manufacturer's instructions. One-step RT-qPCR assays were conducted using the LightMix® Modular Kits (TIB MOLBIOL, Berlin, Germany) complemented with Luna® Universal qPCR Master Mix for amplification and quantitative detection of HCoV RNA. The LightMix® Modular panCoronavirus Kit was used to target the conserved polyprotein gene for the detection of a wide range of coronaviruses, while species specific LightMix® Modular Kits for SARS-CoV-2, HCoV-229E, HCoV-HKU1, HCoV-OC43, and HCoV-NL63 were employed for species confirmation.

Plasma samples were tested using V-PLEX® Coronavirus Panel 3 kits (Meso Scale Discovery, MSD) to quantify immunoglobulin G (IgG) antibody concentrations against the Spike proteins of SARS-CoV-2, SARS-CoV-1, MERS-CoV, HCoV-OC43, HCoV-HKU1, HCoV-NL63, and HCoV-229E, following the manufacturer's instructions. IgG antibodies were selected because they are relatively stable and persist longer following infection or vaccination, making them appropriate markers for assessing prior exposure and population-level seroprevalence. In contrast, antibodies such as IgM and IgA are typically associated with early or acute infection and decline more rapidly over time. Since the primary objective of the serological component of this study was to evaluate cumulative exposure to HCoVs within the study population, IgG was considered the most suitable immunological marker. Cutoff values for positive antibody responses to the SARS-CoV-2 Spike protein were determined from 400 plasma samples collected before the COVID-19 pandemic in the Ashanti and Central Regions of Ghana as described in our previous study (6). Similar cutoff values could not be determined for the other HCoVs as no samples before circulation of HCoV-OC43, HCoV-HKU1, HCoV-NL63, and HCoV-229E were available.

### Data analysis

All statistical analyses were conducted using R (base packages). HCoV RNA prevalence was calculated as the proportion of pan-coronavirus RT-qPCR-positive samples out of the total number of swabs tested, and species-specific prevalence was calculated among pan-coronavirus-positive samples. Serological data were summarized as median IgG antibody concentrations with interquartile ranges. Differences in antibody concentrations between rural and urban populations were assessed using the two-sided Wilcoxon rank sum test, with statistical significance defined as *p* < 0.05. Results were stratified by community and age group to examine population-level patterns of HCoV exposure. Data visualization was performed using the ggplot2 package in R.

### Ethics

Approval for the study was obtained from the Noguchi Memorial Institute for Medical Research (NMIMR) Institutional Review Board (IRB) with federal wide assurance number 00001824, the Ghana Health Service IRB and the Ethics Committee Northwest and Central Switzerland (EKNZ) with Statement ID AO_2023-00020. All individuals above two years of age were eligible for inclusion in the study. Written informed consent was received from all study participants and in the case of minors from legally authorized representatives (parents or guardians) prior to enrollment.

## Results

**Figure 2:**
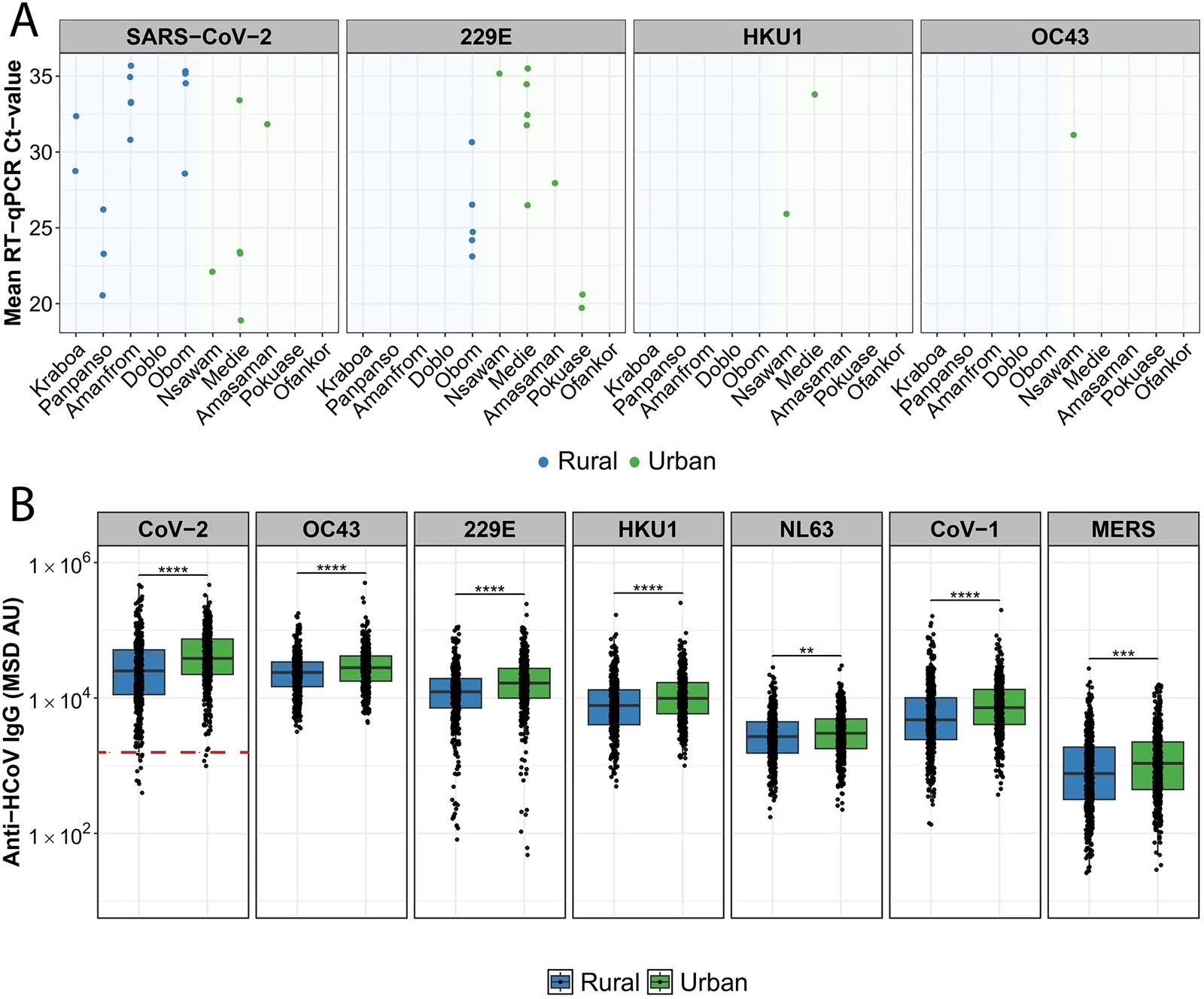
Prevalence of HCoVs in rural and urban Ghana. **(A)** Mean cycle threshold (Ct) values obtained by RT-qPCR for the detection of HCoV RNA in samples from rural (blue) and urban (green) communities. **(B)** Anti-SARS-CoV-2, -OC43, -229E, -HKU1, -NL63, -SARS-CoV-1, and -MERS-CoV Spike antibody concentrations in MSD Arbitrary Units (AU) are depicted for rural (blue) and urban (green) populations. Box-whisker plots show the median and interquartile range as the box and the whisker ends as the most extreme values within 1.5 times the interquartile range below the 25% quantile and above the 75% quantile. Significance between rural and urban groups was tested with two-sided Wilcoxon rank sum test. *p < 0.05, **p < 0.01, ***p < 0.001, ****p < 0.0001. The red dashed line at 1,535 AU/mL indicates the SARS-CoV-2 Spike seropositivity cutoff determined from pre-pandemic plasma samples.

We tested 1,000 naso-/oropharyngeal swabs for the presence of HCoV RNA using a pan-coronavirus RT-qPCR assay. Overall, 6.7% (67/1,000) of samples tested positive, indicating active or very recent coronavirus infection. Among these positives, SARS-CoV-2 was the most frequently detected species (29.9%, 20/67), followed by HCoV-229E (20.9%, 14/67), HCoV-HKU1 (3.0%, 2/67), and HCoV-OC43 (1.5%, 1/67) (Figure 2A). Two participants tested positive for both SARS-CoV-2 and HCoV-229E, indicating co-infection. HCoV-NL63 was not detected. SARS-CoV-2 and HCoV-229E RNA were detected in both rural and urban participants, whereas HCoV-OC43 and HCoV-HKU1 RNA were found only in urban areas. Notably, 47.8% (32/67) of pan-coronavirus-positive samples could not be typed by species-specific RT-qPCR assays.

To assess prior exposure, plasma samples from 989 participants were tested for IgG antibodies against the full-length Spike proteins of SARS-CoV-2, SARS-CoV-1, MERS-CoV, HCoV-229E, HCoV-NL63, HCoV-HKU1, and HCoV-OC43. Antibody responses varied markedly across viruses, with high median concentrations against SARS-CoV-2, HCoV-OC43, and HCoV-229E Spike, and lower median concentrations against HCoV-NL63 and HCoV-HKU1 Spike in both rural and urban populations (Figure 2B). Median antibody concentrations against all five endemic viruses—SARS-CoV-2, HCoV-OC43, HCoV-229E, HCoV-HKU1, and HCoV-NL63—were consistently higher in urban compared to rural populations (Figure 2B). Cross-reactive antibodies to SARS-CoV-1 Spike and, to a lesser extent, MERS-CoV Spike were also detected, reflecting antigenic similarities within the betacoronavirus genus. Based on the defined cutoff, 98.7% of participants showed evidence of prior exposure to SARS-CoV-2 Spike through vaccination or infection (6). The same ranking of antibody levels (SARS-CoV-2 > HCoV-OC43 > HCoV-229E > HCoV-HKU1 > HCoV-NL63) was observed across different communities and age groups (Supplementary Figures 1A,B). Anti-229E and anti-HKU1 antibody concentrations increased with age, while anti-OC43 and anti-NL63 levels remained relatively constant across age groups.

## Discussion

This study provides a contemporary assessment of HCoV circulation in rural and urban Ghana during the post-COVID-19 pandemic period. A substantial proportion of participants (6.7%) had detectable HCoV RNA, indicating ongoing or recent infections within the study population. SARS-CoV-2 and HCoV-229E were the predominant circulating coronaviruses, while HCoV-NL63 was not detected. The presence of co-infections highlights the potential for simultaneous circulation of multiple HCoVs in the population and may complicate respiratory disease surveillance due to overlapping clinical presentations. Notably, nearly half of the pan-coronavirus-positive samples could not be typed using species-specific assays, which may reflect infections with uncharacterized coronaviruses or reduced sensitivity of the species-specific RT-qPCR assays compared to the pan-coronavirus assay. This suggests the importance of incorporating sequencing approaches in the region and in future studies to better define circulating viral diversity. Differences in virus detection between rural and urban settings, with higher diversity observed in urban areas, suggest that population density and mobility may influence transmission dynamics and exposure risk.

There are two main seasons in the study areas—the harmattan/dry and wet seasons. Sampling was conducted between November and December during the harmattan, a period characterized by dry and dusty atmospheric conditions that can affect viral survival and transmission dynamics.

A pre-COVID-19 study (2011–2012) in three rural communities in the Brong Ahafo and Ashanti Regions of Ghana reported a similar HCoV distribution among individuals with acute respiratory tract infections during harmattan (5). In that study, overall HCoV detection rates were higher during the harmattan than in the wet season, with HCoV-229E predominating in harmattan and HCoV-NL63 in the wet season, consistent with our post-pandemic finding of high HCoV-229E prevalence and absence of HCoV-NL63 detection during harmattan, while HCoV-OC43 and HCoV-HKU1 were detected at comparable levels throughout the year.

Seasonal differences suggest that environmental factors, such as humidity and temperatures, which can enhance respiratory virus stability, may influence the prevalence and circulation patterns of specific HCoVs (5). In temperate climates, HCoVs typically show clear seasonal patterns, with most cases occurring in winter (1, 8, 9). Additional investigations are needed to further characterize the seasonality of HCoVs especially in West African countries with tropical climates.

Our serological analysis also demonstrated widespread exposure to HCoVs with particularly high antibody levels against SARS-CoV-2, HCoV-OC43, and HCoV-229E. These findings align with pre-COVID-19 pandemic data from the Brong Ahafo and Ashanti Regions in Ghana reporting HCoV-229E as the most seroprevalent virus, followed by HCoV-OC43 and HCoV-NL63 (10). Higher antibody concentrations in urban populations suggest increased exposure, possibly due to greater population density and interaction rates. The age-related increase in antibody levels for HCoV-229E and HCoV-HKU1 suggests cumulative exposure over time, while stable levels for HCoV-OC43 and HCoV-NL63 may indicate early-life exposure and sustained immunity. The detection of cross-reactive antibodies to SARS-CoV-1 and MERS-CoV reflects antigenic relatedness among coronaviruses, particularly within the betacoronavirus genus.

A limitation of this study is the lack of well-characterized seronegative and positive control sera for the endemic HCoVs as well as for MERS-CoV and SARS-CoV-1, which precluded the determination of virus-specific cutoff values for these assays. Additionally, sampling was restricted to a single season, limiting generalizability across the full annual cycle. Information regarding timing of sample collection relative to symptom onset was also not systematically documented for symptomatic individuals, limiting assessment of associations between viral detection and stage of illness. Future studies should incorporate validated control sera, longitudinal sampling across seasons, and genomic sequencing of pan-coronavirus-positive samples to better define circulating viral diversity, seasonality in tropical West Africa and further refine serological thresholds.

In summary, these findings demonstrate ongoing co-circulation of multiple HCoVs, with SARS-CoV-2 as the predominant strain in both rural and urban Ghana in the post-pandemic era, highlight the influence of environmental and demographic factors on transmission dynamics, and reveal high population-level exposure. These data provide rare epidemiological insight into HCoV circulation in Ghana and offer a baseline level of endemic coronavirus circulation, to guide future surveillance activities.

## Data Availability

The raw data supporting the conclusions of this article will be made available by the authors, without undue reservation.
