# Supplementary material for: High prevalence of human coronaviruses in rural and urban Ghana in the post-COVID-19 era
Source: Front Epidemiol. 2026 Aug 10;6:1874054. doi: 10.3389/fepid.2026.1874054 (PMC13500641; doi:10.3389/fepid.2026.1874054)
Supplement: Supplementary file 1 [file Supplementaryfile1.docx]

**Supplementary Figure 1:** Anti-HCoV IgG antibody concentrations by (A) community and (B) age group. Rural and urban communities are shown in blue and green, respectively.
